# Supplementary material for: Transfer Learning for Alzheimer’s Disease through Neuroimaging Biomarkers: A Systematic Review
Source: Sensors (Basel). 2021 Oct 31;21(21):7259. doi: 10.3390/s21217259 (PMC8587338; doi:10.3390/s21217259)
Supplement: Supplementary file 1 [file sensors-21-07259-s001.zip › sensors-1428588-supplementary.pdf]

Table S1

Acronym	Description
CN	Cognitively Normal
DL	Deep Learning
TL	Transfer Learning
fMRI	functional Magnetic Resonance Imaging
sMRI	Structural Magnetic Resonance Imaging
SAE	Stacked Auto Encoder
SMC	Significant Memory Concern
EMCI	Early mild Cognitive Impairment
MCI	Mild Cognitive Impairment
AD	Alzheimer's Disease
LMCI	Late Mild Cognitive Impairment
HC	Healthy Control
SPM	Statistical Parametric Mapping
CAFFE	Convolutional Architecture for Fast Feature Embedding
SFS	Sequential forward **selection**
CAE	Convolutional Auto Encoder
ICAE	Inception Convolutional Auto Encoder
AIBL	Australian Imaging Biomarkers and Lifestyle Study of Ageing
ADNI	Alzheimer's Disease Neuroimaging Initiative
OASIS	Open Access Series of Imaging Studies
BIDS	Brain Imaging Data Structure Standards
MDNN	Multi-Scale Deep Neural Network
IXI	Information eXtraction from Images (IXI) public dataset
FMRIB	Functional Magnetic Resonance Imaging of the Brain
SPM	Statistical Parametric Mapping
DARETEL	Diffeomorphic Anatomical Registration Exponentiated Lie Algebra

pMCI	Progressive MCI
sMCI	Stable MCI
FDG-PET	Fluorodeoxyglucose positron emission tomography
LDDMM	Large deformation diffeomorphic metric mapping
AV-45 PET	florbetapir positron emission tomography
ILSVRC	ImageNet Large Scale Visual Recognition Challenge
LSTM	Long Short-Term Memory
GRU	Gated Recurrent Unit
DenseNet	Densely Connected Convolutional Networks
SV2A-PET	Synaptic vesicle glycoprotein PET
TSPO-PET	translocator protein PET
FWHM	Full-width at half maximum

Table S2

Ref.	Year	Journal Name	Database	Country	Citations
[68]	2017	International Conference on Bioinformatics and Biomedicine (BIBM)	IEEE Xplore	Canada	19
[59]	2018	ELSEVIER / NeuroImage: Clinical	Science Direct	Saudi Arabia	4
[63]	2018	NCBI/Quantitative imaging in medicine and surgery	PubMed	China	3
[64]	2018	DEStech Transactions/ Computer Science & Engineering	IEEE Xplore	China	1
[65]	2018	ELSEVIER / Medical Image Analysis	Science Direct	Canada	39
[66]	2018	ELSEVIER / Behavioural Brain Research	Science Direct	Korea	55
[58]	2019	Springer/Journal of Medical Systems	Science Direct	Italy	47
[60]	2019	natureresearch/ scientific reports	Web of Science	Korea	12
[69]	2019	ELSEVIER / Journal of Neuroscience Methods	Science Direct	China	10
[62]	2020	ELSEVIER / Journal of Neuroscience Methods	Science Direct	USA	1
[61]	2020	ELSEVIER / Medical Image Analysis	Science Direct	France	1
[67]	2020	ELSEVIER /Saudi Journal of Biological Sciences	Science Direct	China	1
[70]	2020	ELSEVIER / NeuroImage: Clinical	Science Direct	USA	0

Table S3

Ref.	Dataset	Sample size	AD vs. NC	sMCI vs. pMCI	DL Arch.	TL Method	Modality	Data Augmentation	Input	Validation
[58]	ADNI	CN-25,SMC-25,EMCI-25,<br>LMCI-25, MCI-13,AD-25	YES	NO	2d CNN	ResNet-18	rs-fMRI	Yes (3D scans to 2D images)	6160 2D images from each fMRI scan	independent random sample
[59]	ADNI	AD-294,ncMCI-510,<br>cMCI-253,HC-352	YES	YES	3d CNN	Weights of the CNN used for AD vs HC, were transferred to other CNN's and used as initial weights	3 T T1 w images of sMRI	Yes (Augmented the dataset of each subject group up to1000 by using deformation, flipping, scaling, cropping, and rotation of images.)	Normalized 3d T1 -weighted Images	10-fold cross-validation
	MILAN (Local)	AD-124,ncMCI-23,<br>cMCI-27,HC-55								
[60]	ADNI	AD-198,NC-230,<br>pMCI-166,sMCI-101	YES	YES	3d CNN<br>stacked CAE.<br>ICAE	1. Inception module for the convolutional auto encoder-based on GoogleNet.<br>2. A transfer learning scheme for the pMCI classification that transfers the weights learned during AD vs. NC to pMCI vs. sMCI.	1.5T T1 w<br>MP-RAGE<br>sequence<br>of sMRI	Yes (1. Input MRIs are randomly rotated, shifted, and rescaled.<br>2. Intensity changes by randomly adjusting brightness, contrast, saturation, and hue.)	Normalized 3d T1 -weighted Images	Nested 5-fold cross-validation
[61]	ADNI	CN-330,MCI-787, sMCI-298,<br>pMCI-295,AD-336	YES	YES	3d CNN,2d CNN	1. AE pre-training for 3D CNNs<br>2. ResNet pre-trained on ImageNet for 2D CNNs.	1.5T T1w MRI	No(ADNI dataset used for training/validation, test sets were composed of all subjects of OASIS and AIBL)	Normalized T1w MRI	5-fold cross-validation
	OASIS	CN-76,AD-78								
	AIBL	CN-429,MCI-93,sMCI-13,<br>pMCI-20,AD-76								
[62]	ADNI	CN -237, sMCI -245,<br>pMCI -189 ,AD -157	YES	YES	3d CNN	deep ResNet	sMRI	No	Smoothed, Modulated and warped 3D gray matter images	5-fold (stratified) cross-validation
[63]	ADNI	NC-150,sMCI-150,<br>cMCI-157	NO	YES	GoogleNet.<br>CaffeNet	AlexNet (ImageNet)	sMRI	Yes(random views aggregation to generate abundant image patches from the original MR scans)	48 different slices randomly from each subject.	5-fold cross-validation
[64]	ADNI-GO<br>ANDI-2	cMCI-61,ncMCI-276	NO	YES	2d CNN	CaffeNet	sMRI	No	65 slices of each subject	10-fold cross-validation
[65]	ADNI	NC-304,sMCI-409,<br>pMCI-112,AD-226	YES	YES	DNN, SAE	weights of the DNN used for AD vs NC were used in sMCI vs pMCI	18F-FDG-PET + sMRI	No	Multiscale patch-wise metabolism features for each subject	10-fold cross-validation

Ref.	Dataset	Sample size	AD vs. NC	sMCI vs. pMCI	DL Arch.	TL Method	Modality	Data Augmentation	Input	Validation
[66]	ADNI	NC-182,AD-139, cMCI-79,ncMCI-92	YES	YES	Deep CNN	weights of the CNN used for AD vs NC were used in sMCI vs pMCI	18F-FDG and AV-45 PET	Yes(PET images were augmented by flipping in the left-right direction)	Pre-processed images downloaded from ADNI database	10-fold cross-validation
[67]	ADNI	MCIc-70,ncMCI-280	NO	YES	3D CNN	AlexNet (ImageNet)	18F-FDG PET	No	Pre-processed images in LMDB format	No
[68]	OASIS	100-AD,NC-100	YES	NO	3d CNN VGG16 and Inception	AlexNet (ImageNet)	sMRI	No	6400 Images Slices	5-fold cross-validation
[69]	ADNI	AD-194,pMCI-164, sMCI-233,NC-216	YES	YES	DenseNets, RNN	feature learning by DenseNets, used by RNN	3T baseline T1-weighted images of sMRI	Yes(each hippocampus centroid is shifted by ± 2 voxels in x, y and z coordinates to extract more patches.)	3D internal-hippo and external-hippo patches	5-fold cross-validation
[70]	ADNI	NC-256.168-cMCI,129ncMCI	NO	YES	3d CNN	Parameters of the deep network obtained from the pretraining procedures used as it is for the age prediction.	3T baseline T1-weighted images of sMRI	No	Normalized 3d T1 -weighted Images	5-fold cross-validation
	IXI	581-NC								

Table S4

Ref.	Modality	Pre-processing-Pipeline	Category	Software
[58]	rs-fMRI	1. Dataset is transformed from DICOM to NIFTI format. 2. Brain extraction is done on scans.3. Motion correction. 4. Slice timing correction. 5. Intensity normalization 6. Spatial smoothing- Gaussian Kernel (FWHM) 7. High-pass filtering 8. Spatial normalization.	Slice-based	FMRIB Software Library(FSL)
[59]	3 T T1w images of sMRI	1. 3D T1w images from multiple data sources were normalized to the MNI space. 2. Segmentation was performed to yield GM, WM, and CSF tissue probability maps.	Voxel-based	SPM 12
[60]	1.5 T1 w "MP-RAGE" sequence of sMRI	1. T1w scans were rectified for bias-field inhomogeneities .2 Spatial normalization for registering in MNI space and to supervise the suggested visualization process.	Voxel-based	SPM 12
[61]	T1w MRI	1. The N4ITK method is employed for bias field correction. 2. Linear (affine) registration by using the SyN algorithm from ANTs to register each image to the MNI space. 3. Cropping 4.Intensity rescaling.	Voxel-based, ROI-based, Patch-based, Slice-based	Nipype
[62]	sMRI	1. Segmented to identify the GM tissues. 2. Spatial normalization.3.Spatial smoothing - 3D Gaussian kernel to 6 mm (FWHM).	ROI-based	SPM12
[63]	sMRI	1. Gradwarp 2.Intensity inhomogeneity correction 3.N3 histogram peak sharpening.	Slice-based	MATLAB (version R2013a)
[64]	sMRI	1. Segmentation into GM.WM and CSF tissues. 2.Customized template was created based on the GM segmentations by DARTEL.3.GM maps were modulated using the Jacobean determinants.4.Smoothing using an 8-mm FWHM Gaussian kernel.5.Scaling with trilinear interpolation.6.Images were exported in a lossless Portable Network Graphics (PNG) format.7.PCA and sequential feature selection (SFS) were used.	Voxel-based	SPM12
[65]	18F FDG-PET + sMRI	1. sMRI segmented into GM and WM tissues.2.segmentation of ROIs were further subdivided into patches of varying sizes.3. Template MRI was registered to each target MRI space, patch-by-patch with binary segmentation image registration by LDDMM 4. Coregistration of FDG-PET image and skull-stripped MRI scans 5. Average intensities extraction of the FDG-PET image in each patch as the metabolism feature.	ROI-based, Patch-based	FreeSurfer 5.3 package
[66]	18F FDG-PET and AV-45 PET	Already pre-processed PET images were downloaded. 1. Co-registration of FDG and AV-45 PET images .2. Standardized to have the same voxel size.3. Scanner-specific smoothing,	Voxel-based	Preprocessed images were downloaded.
[67]	18F FDG-PET	1. Interpolation. 2. Completion. 3. Segmentation. 4. Segmentation of NifTi image of each subject from the Z-axis into 65 images in PNG format. 5. Grayscale images in the PNG format were converted into pseudo-colored images in the RGB format.6.Image transformation into LMDB format.	Slice-based	NifTi_2014 toolkit
[68]	sMRI	Choose the most explanatory 32 images from the axial plane of each 3D scan by sorting entropies.	Slice-based	MATLAB
[69]	3 T T1w images of sMRI	1. Affine registration .2. Segmentation of the hippocampus and generation of a binary mask for each hippocampus.3.extraction of a fixed size 3D patch from the centre of each hippocampus	ROI-based, Patch-based	FSL
[70]	3 T T1 w images of sMRI	1. Conversion of DICOM files to NIfTI and rigid registeration.2. Images were resampled by employing cubic spline interpolation	Voxel-based	MRIcron
